# Identification of a Novel Ferroptosis-Related Gene Prognostic Signature in Bladder Cancer

**DOI:** 10.3389/fonc.2021.730716

**Published:** 2021-09-07

**Authors:** Jiale Sun, Wenchang Yue, Jiawei You, Xuedong Wei, Yuhua Huang, Zhixin Ling, Jianquan Hou

**Affiliations:** ^1^Department of Urology, The First Affiliated Hospital of Soochow University, Suzhou, China; ^2^Department of Urology, Dushu Lake Hospital Affiliated to Soochow University, Suzhou, China

**Keywords:** ferroptosis, gene signature, bladder cancer, bioinformatics analysis, tumor tissue microarray

## Abstract

**Background:**

Ferroptosis is a newly found non-apoptotic forms of cell death that plays an important role in tumors. However, the prognostic value of ferroptosis-related genes (FRG) in bladder cancer (BLCA) have not been well examined.

**Methods:**

FRG data and clinical information were collected from The Cancer Genome Atlas (TCGA). Then, significantly different FRGs were investigated by functional enrichment analyses. The prognostic FRG signature was identified by univariate cox regression and least absolute shrinkage and selection operator (LASSO) analysis, which was validated in TCGA cohort and Gene Expression Omnibus (GEO) cohort. Subsequently, the nomogram integrating risk scores and clinical parameters were established and evaluated. Additionally, Gene Set Enrichment Analyses (GSEA) was performed to explore the potential molecular mechanisms underlying our prognostic FRG signature. Finally, the expression of three key FRGs was verified in clinical specimens.

**Results:**

Thirty-two significantly different FRGs were identified from TCGA–BLCA cohort. Enrichment analyses showed that these genes were mainly related to the ferroptosis. Seven genes (TFRC, G6PD, SLC38A1, ZEB1, SCD, SRC, and PRDX6) were then identified to develop a prognostic signature. The Kaplan–Meier analysis confirmed the predictive value of the signature for overall survival (OS) in both TCGA and GEO cohort. A nomogram integrating age and risk scores was established and demonstrated high predictive accuracy, which was validated through calibration curves and receiver operating characteristic (ROC) curve [area under the curve (AUC) = 0.690]. GSEA showed that molecular alteration in the high- or low-risk group was closely associated with ferroptosis. Finally, experimental results confirmed the expression of SCD, SRC, and PRDX6 in BLCA.

**Conclusion:**

Herein, we identified a novel FRG prognostic signature that maybe involved in BLCA. It showed high values in predicting OS, and targeting these FRGs may be an alternative for BLCA treatment. Further experimental studies are warranted to uncover the mechanisms that these FRGs mediate BLCA progression.

## Introduction

Bladder cancer (BLCA) is the second most frequently diagnosed urinary cancer, with an estimated 573,000 new cases and 213,000 deaths worldwide in 2020 (1). As it is a heterogeneous disease with various great challenges (2), patients with BLCA often suffer from high rates of tumor recurrence, progression, and metastasis. The 5-year relapse-free survival of non-muscle-invasive BLCA ranges from 23% to 43% and 5-year overall survival of muscle invasive BLCA ranges from 36% to 48% (3, 4). Moreover, universally recognized BLCA prognostic tissue biomarkers are still lacking (5), making it difficult for urologists to stratify the risk and determine the precise treatment decision on patients with BLCA. In the past decade, gene signature, which is a combined group of genes in a cell or tissue (6), has been widely used for risk stratification of patients with cancer (7). Meanwhile, with the rapid development of network-performed genomic studies including The Cancer Genome Atlas (TCGA) and Gene Expression Omnibus (GEO), detailed characterization of genetic and epigenetic alterations of cancer could be provided, fundamentally transforming our view of cancer biology (8). Therefore, *via* data mining and bioinformatics analysis, it is feasible to develop an accurate survival risk stratification model based on gene signature for patients with BLCA.

Ferroptosis, which was first introduced in 2012, is a newly found non-apoptotic forms of cell death that occurs through excessive peroxidation of polyunsaturated fatty acids (9). Emerging evidence shows that ferroptosis can inhibit the development of various tumors and may be beneficial for cancer treatment (10). Up to now, a large number of genes have been confirmed to be involved in the initiation and execution of ferroptosis in cancer, such as p53, GPX4, and NRF2 (11–13). In addition, several drugs, which have the ability to induce ferroptosis in cancer cells, have been approved by the Food and Drug Administration (FDA), and new compounds are still being found by researchers (14). In short, ferroptosis should be a promising area in cancer research. However, to our best knowledge, studies exploring the association between ferroptosis−related genes (FRGs) and BLCA, and their relationships with survival in patients with BLCA are still lacking.

In this study, we aimed to thoroughly investigate the role of FRG in BLCA and develop a novel survival risk stratification model based on FRG signature. First, by retrieving RNA-sequencing (RNA-seq) data from TCGA database, FRG expression profiles and their values in the prognosis in BLCA were comprehensively analyzed. Subsequently, an FRG signature in TCGA cohort was established and then validated in the GEO cohort. Finally, three of the potential key genes in our FRG signature were validated in clinical specimens by immunohistochemistry (IHC) staining *via* tumor tissue microarray (TMA). We expect that our findings will give a more comprehensive insight into the role of FRG in BLCA.

## Methods and Materials

### Acquisition and Preprocess of Public Data

The BLCA RNA-seq transcriptome profiles and related clinical information were downloaded from TCGA (https://portal.gdc.cancer.gov/). Besides, one BLCA GEO cohorts with detailed clinical data were downloaded, namely, GSE13507. The GEO database is an international public repository that archives and freely distributes high-throughput gene expression and other functional genomics datasets. Next, a total of 149 validated FRGs were identified through the FerrDb database (http://www.zhounan.org/ferrdb/) (15) and further analyzed. FerrDb database is the world’s first manually curated database for ferroptosis. A total of 784 ferroptosis articles were downloaded from the PubMed database, and ferroptosis regulators and markers and associated diseases were extracted and annotated, which means that the relationship between these genes and ferroptosis are confirmed by high-quality published studies. In summary, 253 regulators (including 108 drivers, 69 suppressors, 35 inducers, and 41 inhibitors), 111 markers, and 95 ferroptosis–disease associations were included in this database. As the different confidence levels were set to indicate FRGs reliability in FerrDb database, confidence of “validated” (the correlation with ferroptosis was confirmed by experimental studies) was our criterion for FRG selection in this study. The detailed information is available in **Supplementary Material 1**.

### Identification of Significantly Different FRGs and Enrichment Analysis

The “limma” R package was used to identify differentially expressed FRGs with a cutoff value set at |log_2_fold change| > 0.5 and a false discovery rate (FDR) <0.05. Gene Ontology (GO) (16) and Kyoto Encyclopedia of Genes and Genomes (KEGG) pathway enrichment analyses (17) of the differentially expressed FRGs were performed using the “clusterProfiler” R package, and top 10 enrichment terms were exhibited in this study.

### Development and Validation of FRG Prognostic Signature

Univariate cox regressions were used to evaluate the relationships between the differentially expressed FRGs and the patients’ overall survival (OS) in TCGA. All genes with p < 0.1 were screened for further analysis. Subsequently, the least absolute shrinkage and selection operator (LASSO) analysis was performed to select optimal prognostic genes related to OS. The risk score was calculated by the following formula: $\mathrm{risk score}=\Sigma_{n=1}^{j}\;\text{Coef j}*\text{Xj},$ with Coef j referring to coefficient calculated by LASSO and X_j_ referring to messenger RNA (mRNA) expression of FRGs. Then, patients were divided into high- and low-risk groups according to the above risk score. The performance of FRG signature was assessed using Kaplan–Meier analysis. Finally, GSE13507 dataset was used for validating the predictive ability of this signature.

### Establishment of Nomogram

The univariate and multivariate cox regression analyses were performed to identify whether the risk scores and relevant clinical parameters could be predictors associated with OS for patients with BLCA. The performance of risk score and relevant clinical parameters were evaluated by receiver operating characteristic (ROC) curve. Next, a prognostic nomogram was generated to predict 1-, 3-, and 5-year OS of patients with BLCA in TCGA. Internal validation of the model was tested using bootstrap resamples, and the calibration plot was showed graphically.

### Gene Set Enrichment Analyses

To further explore the potential molecular mechanisms underlying our prognostic FRG signature, Gene Set Enrichment Analyses (GSEA) (18) was performed using the “gsva” R package. Top 5 enrichment terms in high- or low-risk group were exhibited in this study.

### Exploration of Key Genes in FRG Signature and Experimental Validation

The Gene Expression Profiling Interactive Analysis (GEPIA2) database (http://gepia2.cancer-pku.cn/) (19) was applied to plot survival curves and boxplots of all genes in FRG signature. Then, we selected three potential key genes (SCD, SRC, and PRDX6) and verified their expression profiles in BLCA *via* TMA slide (HBlaU060CS02), which was obtained from Outdo Biotech Co. Ltd. (Shanghai, China). The slide contained 60 samples (30 tumor tissues and 30 paired adjacent normal tissues) from 30 patients with stage I–IV BLCA. Next, IHC staining was performed directly on this slide. Twenty-nine of tumor tissues were selected for further analysis, as one was exfoliated. The primary antibodies were as follows: anti-SCD (23393-1-AP, Proteintech), anti-SRC (11097-1-AP, Proteintech), and anti-PRDX6 (13585-1-AP, Proteintech). Antibody staining was visualized with 3,3′-diaminobenzidine (DAB) and hematoxylin counterstain. Ethical approval for the study of TMA was granted by the Clinical Research Ethics Committee, Outdo Biotech (Shanghai, China).

### Evaluation of IHC Staining

The TMA slides were digitally scanned using Aperio Digital Pathology Slide Scanners. All results of IHC were evaluated using an established semiquantitative approach by two independent pathologists in a blind manner. If the evaluations were different between two pathologists, the sections were reviewed jointly, and consensus results were obtained. If disagreement still existed, a senior pathologist would be invited to arbitrate. According to the intensity of the staining, the positive reaction of SCD, SRC, and PRDX6 were scored into four grades: 0 (negative), 1 (low), 2 (moderate), and 3 (high). The percentages of SCD-, SRC-, and PRDX6-positive cells were also scored into five grades: 0 (0%), 1 (<10%), 2 (10%–50%), 3 (51%–80%), and 4 (>80%). The immunoreactive score (IRS) gives a range of 0–12 as a product of multiplication between the intensity and percentage scores.

### Statistical Analysis

All statistical analysis and figures exhibition were implemented using R 4.0.3 (R Foundation, Vienna, Austria). Continuous variables of two groups were analyzed with Student’s t-test or Mann–Whitney U-test. Log-rank test was performed to assess the difference between the survival curves. A hazard ratio (HR) and a 95% confidence interval (CI) were evaluated by univariable and multivariate Cox regression models. If not specified above, p < 0.05 was considered statistically significant.

## Results

### Identification of Differentially Expressed FRGs in TCGA Cohort

**Figure 1:**
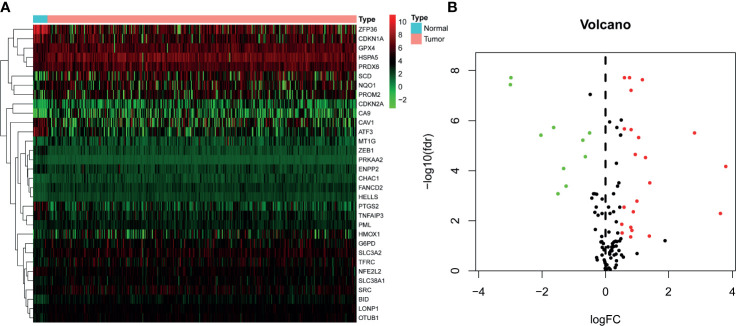
Identification of differentially expressed FRGs in TCGA–BLCA cohort. **(A)** Heatmap of FRGs. Green represents downregulation, and red represents upregulation of genes. **(B)** Volcano plot of FRGs. Green dots represent 10 downregulated genes; red dots represent 22 upregulated genes. FRG, ferroptosis−related gene; TCGA, The Cancer Genome Atlas; BLCA, bladder cancer.

A total of 402 BLCA samples and 19 normal samples with gene expression profiles and clinical information were retrieved from TCGA dataset. After analysis, 32 of 149 FRGs were identified significantly different between normal and BLCA samples (**Supplementary Material 2**). Among these, 10 FRGs were downregulated in BLCA, and 22 genes were upregulated. The heatmap and volcano plots are shown in **Figures 1A, B**.

### Functional Enrichment Analysis of Differentially Expressed FRGs

**Figure 2:**
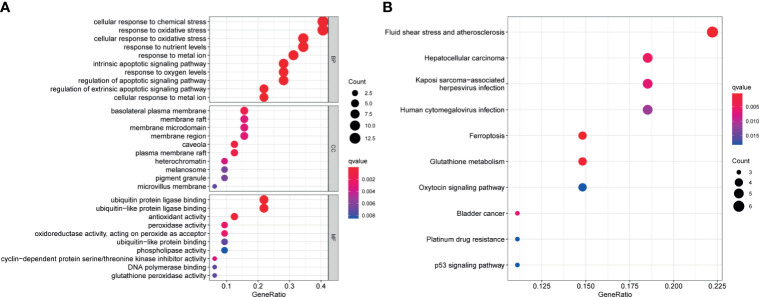
Functional enrichment analysis of the 32 differentially expressed FRGs in TCGA-BLCA cohort. Top 10 enriched biological processes, molecular functions, cellular components **(A)**, and KEGG pathways **(B)** terms of 32 differentially expressed FRGs are shown in this study. FRG, ferroptosis−related gene; TCGA, The Cancer Genome Atlas; BLCA, bladder cancer; KEGG, Kyoto Encyclopedia of Genes and Genomes.

Functional enrichment analysis was used to explore the biological functions and pathways of the above 32 FRGs in TCGA–BLCA cohort. The GO results showed that these FRGs were enriched in iron-related terms, such as response to metal ion and oxidative process (**Figure 2A**). KEGG analysis also showed that these FRGs were closely enriched in ferroptosis and bladder cancer (**Figure 2B**).

### Development of FRG Prognostic Signature in TCGA Cohort

**Figure 3:**
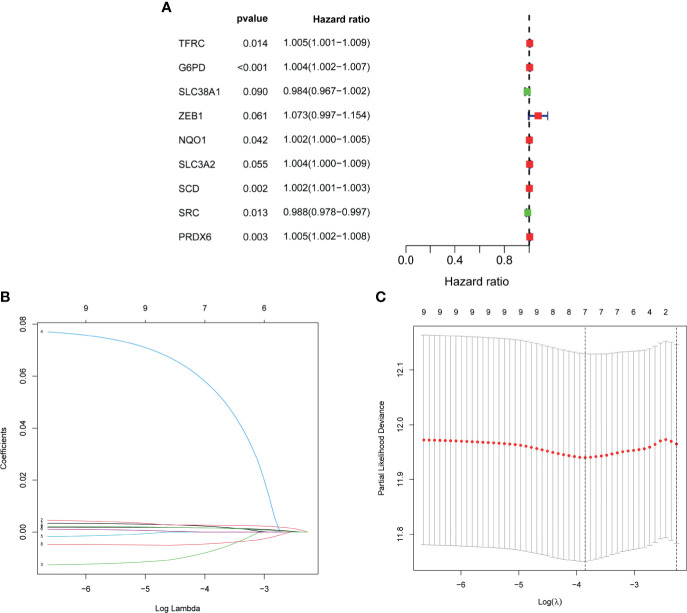
Development of FRG prognostic signature in TCGA cohort. **(A)** Results of the univariate cox analysis of the OS in TCGA-BLCA cohort. **(B)** Ten-time cross-validation for tuning parameter selection in the LASSO Cox regression model. **(C)** LASSO coefficient profiles of the seven ferroptosis-related genes. FRG, ferroptosis−related gene; TCGA, The Cancer Genome Atlas; BLCA, bladder cancer; LASSO, least absolute shrinkage and selection operator.

**Figure 4:**
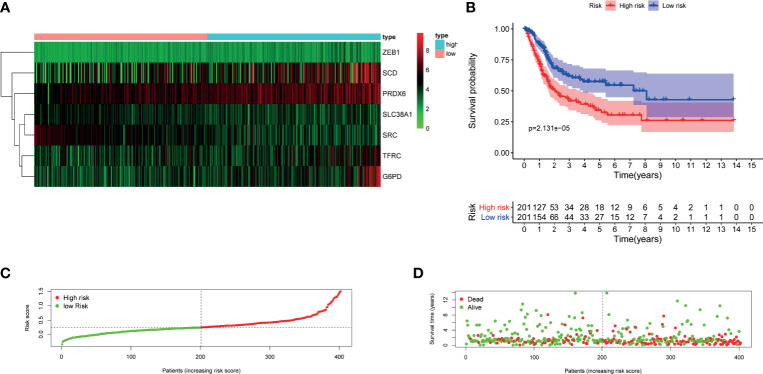
Evaluation of the constructed FRG prognostic signature in TCGA–BLCA cohort. **(A)** Heatmap of seven genes expression between high- and low-risk score group in TCGA–BLCA cohort. **(B)** Kaplan–Meier survival curve. **(C)** Risk score curve plot. The dotted line indicates the individual distribution of risk score, and the patients are categorized into low-risk (green) and high-risk (red) groups. **(D)** Risk score scatter plot. Red dots indicate the dead patients, and green dots indicate the alive. With the increase in risk score, more patients died. FRG, ferroptosis−related gene; TCGA, The Cancer Genome Atlas; BLCA, bladder cancer.

Univariate cox regression analysis was first performed to analyze the above 32 FRGs. The results showed that nine FRGs are correlated with OS of patients with BLCA (p < 0.1) (**Figure 3A**). Next, LASSO analysis was performed to select optimal prognostic genes related to OS (**Figures 3B, C**). As a result, a total of seven genes (TFRC, G6PD, SLC38A1, ZEB1, SCD, SRC, and PRDX6) were identified and were selected to develop a prognostic signature. The risk score = (0.00144 × expression of TFRC) + (0.00255 × expression of G6PD) + (−0.00415 × expression of SLC38A1) + (0.04419 × expression of ZEB1) + (0.001382 × expression of SCD) + (−0.00382 × expression of SRC) + (0.001288 × expression of PRDX6). According to the median risk score, patients were divided into high- and low-risk groups. The heatmap of seven genes expression between high- and low-risk score groups in TCGA cohort was shown in **Figure 4A**. The survival curve demonstrated that the high-risk group showed a poor overall survival compared to low-risk group (**Figures 4B–D**).

### Validation of FRG Prognostic Signature in GEO Cohort

**Figure 5:**
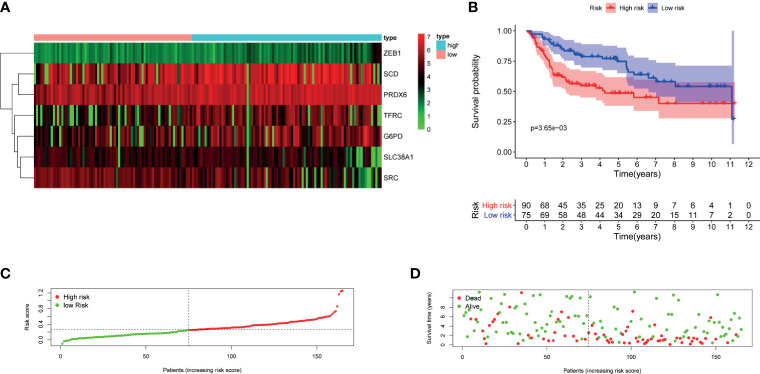
Validation of the constructed FRG prognostic signature in GSE13507. **(A)** Heatmap of seven genes expression between high- and low-risk score group in GSE13507 cohort. **(B)** Kaplan–Meier survival curve. **(C)** Risk score curve plot. The dotted line indicates the individual distribution of risk score, and the patients are categorized into low-risk (green) and high-risk (red) groups. **(D)** Risk score scatter plot. Red dots indicate the dead patients, and green dots indicate the alive. With the increase in risk score, more patients died. FRG, ferroptosis−related gene.

To validate the performance of the FRG signature in predicting OS, risk scores were calculated with the same formula for patients in GSE13507. The heatmap of seven genes expression between high- and low-risk score group in GEO cohort was is in **Figure 5A**. Similarly, the survival curve in GEO cohort also demonstrated that the high-risk group showed a poor overall survival compared to the low-risk group (**Figures 5B–D**).

### Establishment of Nomogram

**Figure 6:**
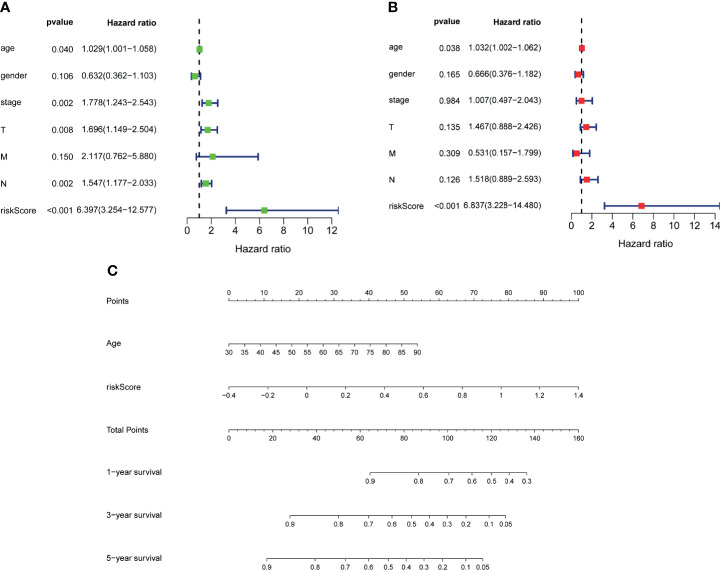
Establishment of nomogram. Univariate **(A)** and multivariate **(B)** analyses assessing relationship between risk scores and relevant clinical parameters and OS in TCGA–BLCA cohort. **(C)** Establishing of a signature-based prognostic nomogram predicting OS in BLCA. OS, overall survival; TCGA, The Cancer Genome Atlas; BLCA, bladder cancer.

**Figure 7:**
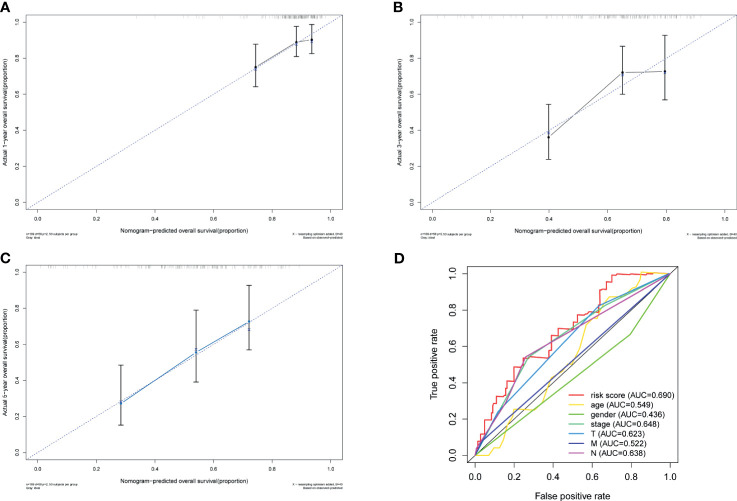
Validation of nomogram. Calibration curves of the nomogram prediction of 1-year **(A)**, 3-year **(B)**, and 5-year **(C)** OS of patients in TCGA–BLCA cohort. **(D)** ROC curve of the risk score and other relevant clinical parameters. OS, overall survival; TCGA, The Cancer Genome Atlas; BLCA, bladder cancer; ROC, receiver operating characteristic.

To further explore the accuracy of this signature, we investigated whether it could work as an independent prognostic factor for OS of TCGA–BLCA cohort. Risk score and clinical traits were included in the univariate and multivariate analyses. Univariable cox regression analysis revealed that age, stage, T, N, and risk score were significantly correlated with OS (p < 0.05) (**Figure 6A**). Next, based on the multivariate analysis, age and risk score were still confirmed as independent predictors for OS (p < 0.05) (**Figure 6B**). Then, the above two variables were used to construct the nomogram for OS (**Figure 6C**). The calibration curves exhibited high consistency between the actual proportion of 1- and 5-year OS and the nomogram-predicted probability (**Figures 7A–C**). Finally, the result of the ROC curve showed that the risk score had better predictive ability compared to other relevant clinical parameters (AUC = 0.690) (**Figure 7D**).

### Gene Set Enrichment Analysis

**Figure 8:**
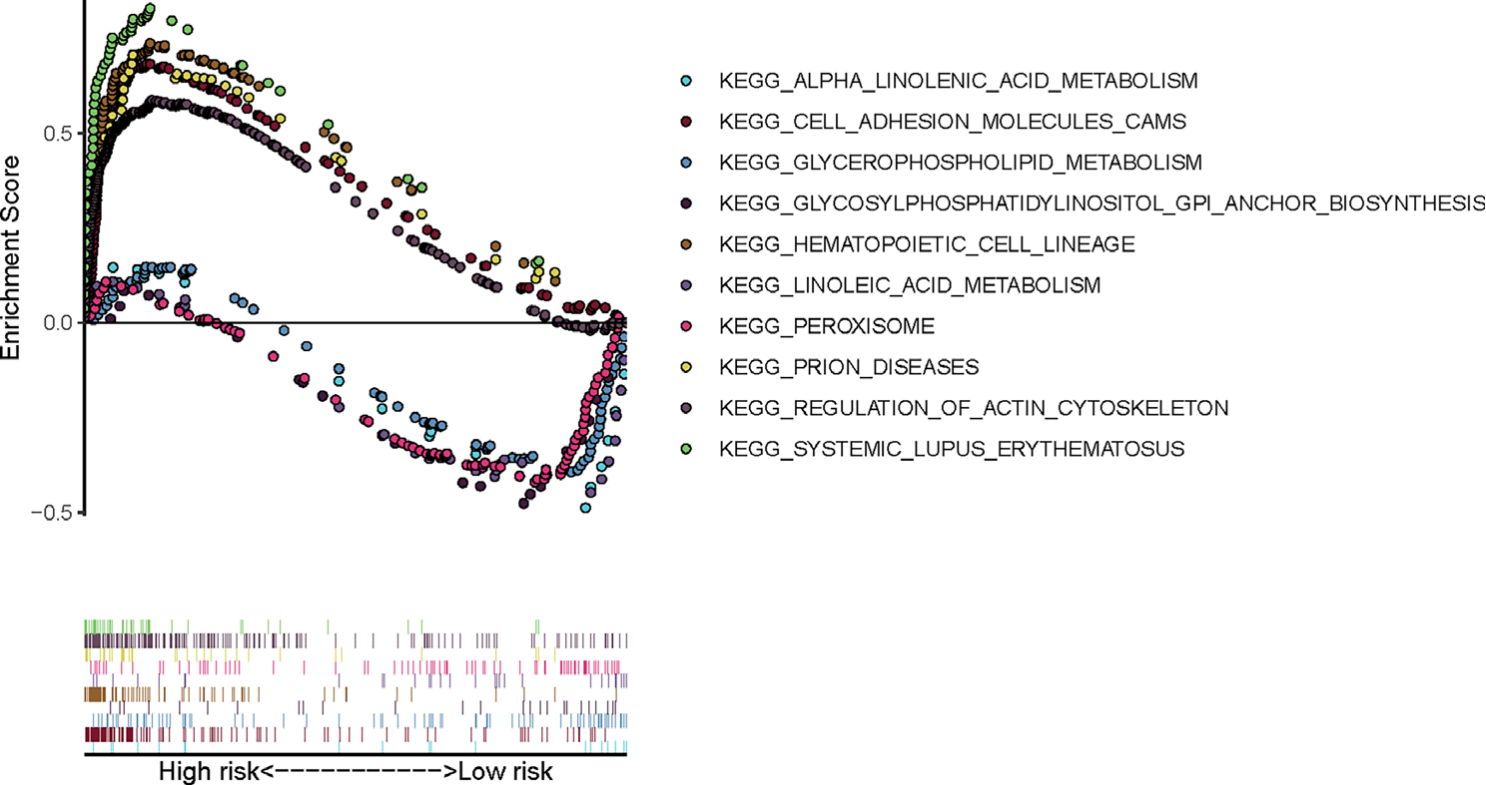
Gene Set Enrichment Analysis of our prognostic FRG signature in TCGA-BLCA cohort. FRG, ferroptosis−related gene; TCGA, The Cancer Genome Atlas; BLCA, bladder cancer.

GSEA was performed to further explore the potential molecular mechanisms underlying our prognostic FRG signature in TCGA cohort. As is shown in **Figure 8**, top 5 enrichment KEGG terms in high- or low-risk group were exhibited, including linolenic acid metabolism and peroxisome, which indicated that molecular alteration in the high- or low-risk group was closely associated with ferroptosis.

### Clinical Experimental Validation

**Figure 9:**
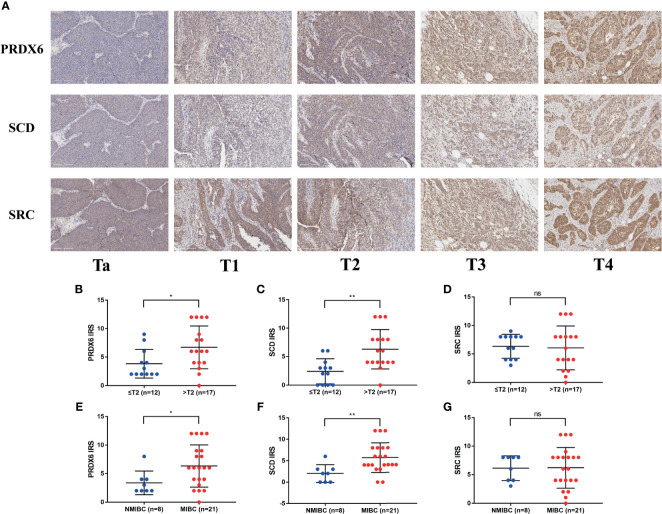
Immunohistochemistry results of three key FRGs. **(A)** Representative immunohistochemistry images of PRDX6, SCD, and SRC in different T stage of BLCA. Comparisons of IRS of PRDX6 **(B, E)**, SCD **(C, F)**, and SRC **(D, G)** between NMIBC and MIBC or in different T stage of BLCA. *p < 0.05, **p < 0.01, ns means no significant difference. FRG, ferroptosis−related gene; BLCA, bladder cancer; IRS, immunoreactive score; NMIBC, non-muscle invasive bladder cancer; MIBC, muscle-invasive bladder cancer.

Before performing IHC validation in clinical specimens, we used the GEPIA2 database to plot survival curves and boxplots of all genes in our FRG signature (**Supplementary Materials 3**, **4**). The Kaplan–Meier curves showed that BLCA patients with three aberrant genes expression (SCD, SRC, and PRDX6) exhibited significantly different OS, respectively (p < 0.05). Therefore, SCD, SRC, and PRDX6 were considered to be the key prognostic genes, and their expression profiles were further verified by IHC *via* TMA slide, respectively. In this study, a total of 10 evaluations did not reach a consensus. As mentioned above, all these sections were reviewed jointly, and six consensus results were obtained. For the remaining four, a senior pathologist was invited to arbitrate. All detailed clinical information of patients is shown in **Supplementary Material 5**, and overview of IHC results of PRDX6, SCD, and SRC in TMA slides are shown in **Supplementary Materials 6**–**8**, respectively. Representative immunohistochemistry images of PRDX6, SCD, and SRC in different T stages of BLCA are shown in **Figure 9A**. Next, analysis using IRS suggested that patients with advanced T stage tended to express higher PRDX6 (**Figure 9B**) (p < 0.05) and SCD (**Figure 9C**) (p < 0.01) but not SRC (**Figure 9D**) (p > 0.05). Similarly, compared with non-muscle invasive bladder cancer (NMIBC), samples with muscle-invasive bladder cancer (MIBC) expressed higher PRDX6 (**Figure 9E**) (p < 0.05) and SCD (**Figure 9F**) (p < 0.01) but not SRC (**Figure 9G**) (p > 0.05). Therefore, our IHC results indicate that PRDX6 and SCD might be the two most significant FRGs that are correlated with malignancy potential in BLCA.

## Discussion

**Figure 10:**
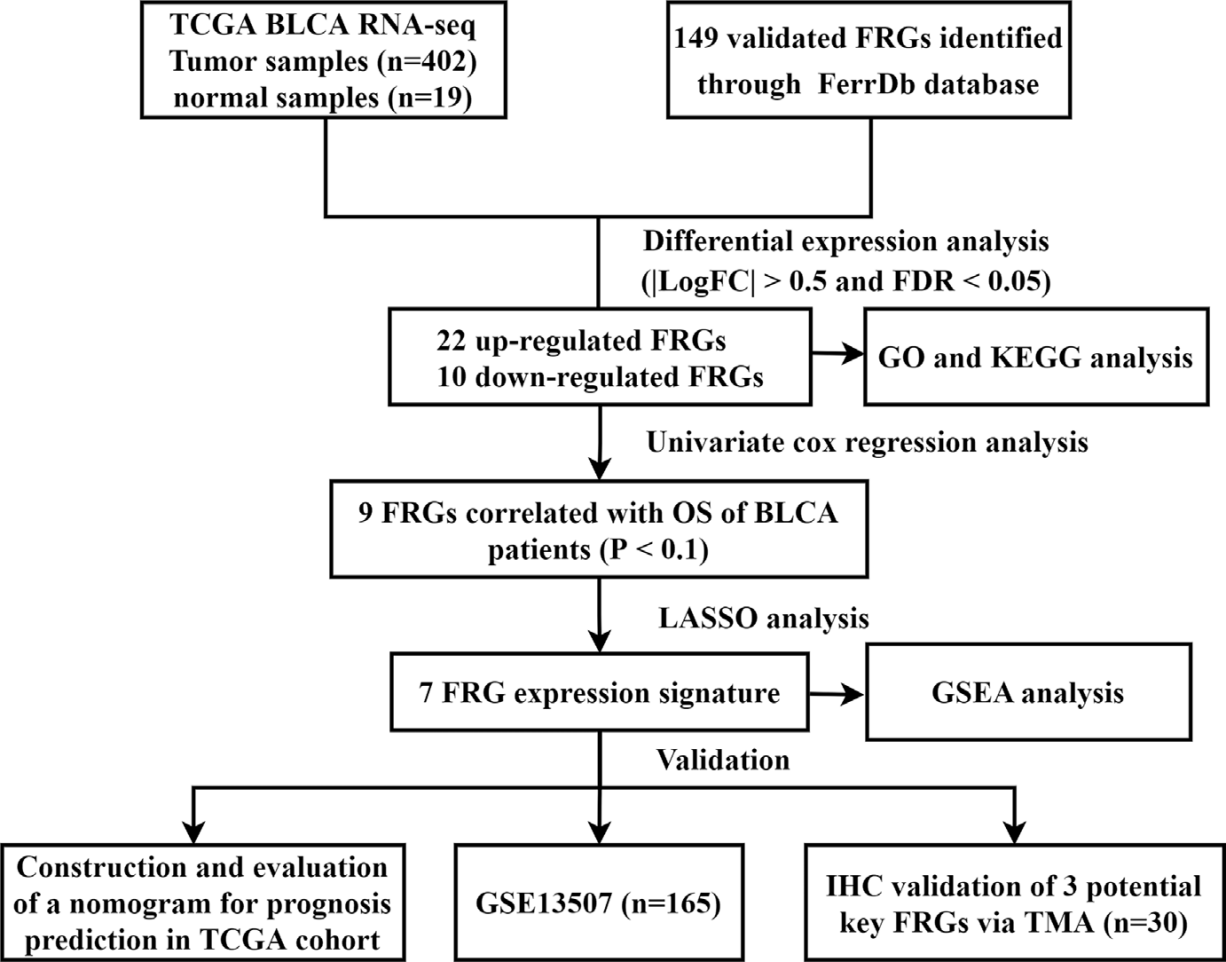
Flow chart of data collection and analysis. TCGA, The Cancer Genome Atlas; BLCA, bladder cancer; FRG, ferroptosis−related gene; GO, gene ontology; KEGG, Kyoto Encyclopedia of Genes and Genomes; OS, overall survival; LASSO, least absolute shrinkage and selection operator; GSEA, Gene Set Enrichment Analysis; IHC, immunohistochemistry; TMA, tissue microarray.

In this study, we collected gene expression data and clinical information of BLCA from public database. A total of 32 FRGs were identified, of which 7 genes were prognostic FRGs by LASSO analysis. Subsequently, a seven-gene signature was constructed, which demonstrated high predictive accuracy. We also developed a nomogram integrating clinical traits and risk scores. The prognostic accuracy of the nomogram was confirmed by the ROC curve and calibration plots. GSEA also indicated that molecular alteration in the high- or low-risk group was closely associated with ferroptosis. Last but not the least, the results of IHC *via* TMA validated the role of three key genes in our FRG prognostic model. Taken together, these findings strongly implied the great potential roles of ferroptosis in BLCA (**Figure 10**).

Liu et al. (20) previously constructed a FRG signature model to predict OS in BLCA. However, only 60 FRGs were included in their study compared to 149 FRGs in our study. Another limitation of their study was the lack of experimental validations. Therefore, we believe that the conclusion based on this study is more convincing. Meanwhile, several FRG signature models for predicting prognosis of other types of cancer have also been established so far. Liu et al. (21) developed a prediction signature including FDFT1, DUOX1, ALOX12B, ATG13, CAV1, NOS2, JDP2, DRD4, TFAP2C, and PLIN4 in colorectal cancer. They also generated a genomic–clinicopathological nomogram integrating age, stage, and risk scores and demonstrated high predictive accuracy. Zhu et al. (22) constructed the predictive model composed of four FRGs in esophageal adenocarcinoma, which had good predictive ability (AUC = 0.744). Furthermore, they performed the polymerase chain reaction and IHC validation in clinical specimens to verify significantly different genes expression profiles in esophageal adenocarcinoma and normal tissues. In clear cell renal cell carcinoma, Wu et al. (23) built a new survival model based on five risk-related FRGs (CARS, NCOA4, FANCD2, HMGCR, and SLC7A11), which showed strong association with clinicopathological features of patients. In addition, Jiang et al. (24) revealed that FRG signature had relevance with the immune characteristics, which may help improve the efficacy of personalized immunotherapy in pancreatic cancer. Collectively, the above findings all indicated the importance of exploring novel FRGs in cancer.

The prognostic model proposed in the present study was composed of seven FRGs (TFRC, G6PD, SLC38A1, ZEB1, SCD, SRC, and PRDX6). Of them, SCD, SRC, and PRDX6 were considered to be the key genes in our FRG signature, which were further verified by IHC in our study. SCD is a lipid-modifying enzyme that catalyzes the desaturation of saturated fatty acids (25), and previous studies have reported that SCD upregulated in numerous malignancies (26–28). Moreover, Tesfay et al. (29) demonstrated that it could protect ovarian cancer cells from ferroptotic cell death. They observed that inhibition of SCD significantly potentiated the antitumor effect of ferroptosis inducers. Ye et al. (30) reported that SCD inhibited both ferroptosis and apoptosis in pancreatic cancer cells. In our study, we verified the aberrant expression of SCD in BLCA tissues and found that samples with high T stage or MIBC expressed higher SCD, which assumed that SCD may play an important oncogenic role in BLCA. SRC is an indispensable player of multiple physiological homeostatic pathways (31). It was also reported that SRC activation could contribute to ferroptosis resistance (32). In our study, we found that the expression of SRC were higher in BLCA than normal tissues in TCGA cohort. However, those with lower level of SRC showed a poor OS compared to those with high level. Interestingly, the expression level of SRC were not consistent with the result of the survival plot in TCGA cohort. The result of TMA in our study showed that the protein expression of SRC showed no significant difference between patients with NMIBC and those with MIBC or in different T stage. It might be due to the relatively small number of BLCA samples included in our study, and further experimental studies are warranted to identify the underlying mechanism. PRDX6, a member of peroxiredoxins (PRDXs), is thought to catalyze the reduction in cellular peroxides to protect cells against oxidative damage (33). Hu et al. (34) reported that PRDX6 could promote the proliferation, migration, and invasion and inhibited apoptosis in cervical cancer cells. Recently, PRDX6 has been also revealed to be essential in protecting cells against ferroptosis (35, 36). In line with the above studies, our result also suggested that PRDX6 could play an oncogenic role in BLCA.

Candidate biomarkers in BLCA are abundant, but few have been validated in clinical practice (37). In our study, we constructed a novel survival risk stratification model based on FRG signature and verified three FRGs expression in clinical samples. What is more, our experimental results preliminarily confirmed the oncogenic role of two FRGs, PRDX6, and SCD, in our signature, which were not reported in BLCA previously. Therefore, the present findings may provide new insight into the precision treatment in BLCA, which involves tumor-specific and ferroptosis−related biomarkers.

The study has several limitations. First, the underlying mechanism how these FRGs in our prognostic signature modulates the process of BLCA is still unclear. Their biological function requires further exploration with well-designed experiments. Second, the present model was both developed and validated with retrospective data from public database. More data based on prospective studies are still needed to verify its clinical utility. Lastly, ferroptosis is a newly rising area in cancer research. As more and more FRGs will be discovered in the future, genes that were identified in this study may be incomplete, meaning that the update of this FRG prognostic signature in BLCA is of great significance.

## Conclusion

In conclusion, we identified seven FRGs that may play an important role in the process of BLCA. The prognostic signature based on these genes demonstrated good accuracy in predicting the patients’ OS and may be promising therapeutic targets. Further experimental studies are warranted to uncover the potential mechanisms that these FRGs mediate BLCA progression.

## Data Availability Statement

The RNA sequencing data and related clinical information were obtained from TCGA (http://cancergenome.nih.gov/) databases and GEO databases (https://www.ncbi.nlm.nih.gov/geo/query/acc.cgi?acc=GSE13507). All other data and materials are available from the corresponding author upon reasonable request.

## Ethics Statement

The studies involving human participants were reviewed and approved by Clinical Research Ethics Committee, Outdo Biotech (Shanghai, China). The patients/participants provided their written informed consent to participate in this study. Written informed consent was obtained from the individual(s) for the publication of any potentially identifiable images or data included in this article.

## Author Contributions

JH and ZL conceived the idea. ZL and JS designed the study. JS, XW, JY, and WY collected the data and performed the experiments. JS drafted the manuscript. ZL and JH reviewed and corrected the manuscript. All authors contributed to the article and approved the submitted version.

## Funding

This work was supported by grants from the National Natural Science Foundation of China (Nos. 82002715, 81472401, and 81772708), Natural Science Foundation of Jiangsu Province (BK20190170), and Programs for Recruitment of Clinical Medical Top Team of Suzhou.

## Conflict of Interest

The authors declare that the research was conducted in the absence of any commercial or financial relationships that could be construed as a potential conflict of interest.

## Publisher’s Note

All claims expressed in this article are solely those of the authors and do not necessarily represent those of their affiliated organizations, or those of the publisher, the editors and the reviewers. Any product that may be evaluated in this article, or claim that may be made by its manufacturer, is not guaranteed or endorsed by the publisher.

## Supplementary Material

Supplementary 1 Detailed information of 149 FRGs identified through the FerrDb database. FRG, ferroptosis−related gene.

Supplementary 2 Significantly different FRG expression levels in BLCA and normal tissue in TCGA cohort. FRG, ferroptosis−related gene; TCGA, The Cancer Genome Atlas; BLCA, bladder cancer.

Supplementary 3 Boxplots of all genes in our FRG signature. FRG, ferroptosis−related gene.

Supplementary 4 Survival curves of all genes in our FRG signature. FRG, ferroptosis−related gene.

Supplementary 5 Detailed clinical information of patients with BLCA.

Supplementary 6 Immunohistochemistry results of PRDX6 in tissue microarray.

Supplementary 7 Immunohistochemistry results of SCD in tissue microarray.

Supplementary 8 Immunohistochemistry results of SRC in tissue microarray.

## Abbreviations

BLCA, bladder cancer; TCGA, The Cancer Genome Atlas; GEO, Gene Expression Omnibus; FRG, ferroptosis−related genes; RNA-seq, RNA sequencing; IHC, immunohistochemistry staining; TMA, tumor tissue microarray; FDR, false discovery rate; GO, Gene Ontology; KEGG, Kyoto Encyclopedia of Genes and Genomes; OS, overall survival; LASSO, least absolute shrinkage and selection operator; ROC, receiver operating characteristic; GSEA, Gene Set Enrichment Analyses; GEPIA2, The Gene Expression Profiling Interactive Analysis; IRS, immunoreactive score; HR, hazard ratio; CI, confidence interval; AUC, area under curve; NMIBC, non-muscle invasive bladder cancer; MIBC, muscle-invasive bladder cancer.
