# Supplementary material for: Identification of a Novel Ferroptosis-Related Gene Prognostic Signature in Bladder Cancer
Source: Front Oncol. 2021 Sep 7;11:730716. doi: 10.3389/fonc.2021.730716 (PMC8455063; doi:10.3389/fonc.2021.730716)
Supplement: Supplementary 1 — Detailed information of 149 FRGs identified through the FerrDb database. FRG, ferroptosis−related gene. [file DataSheet_1.pdf]

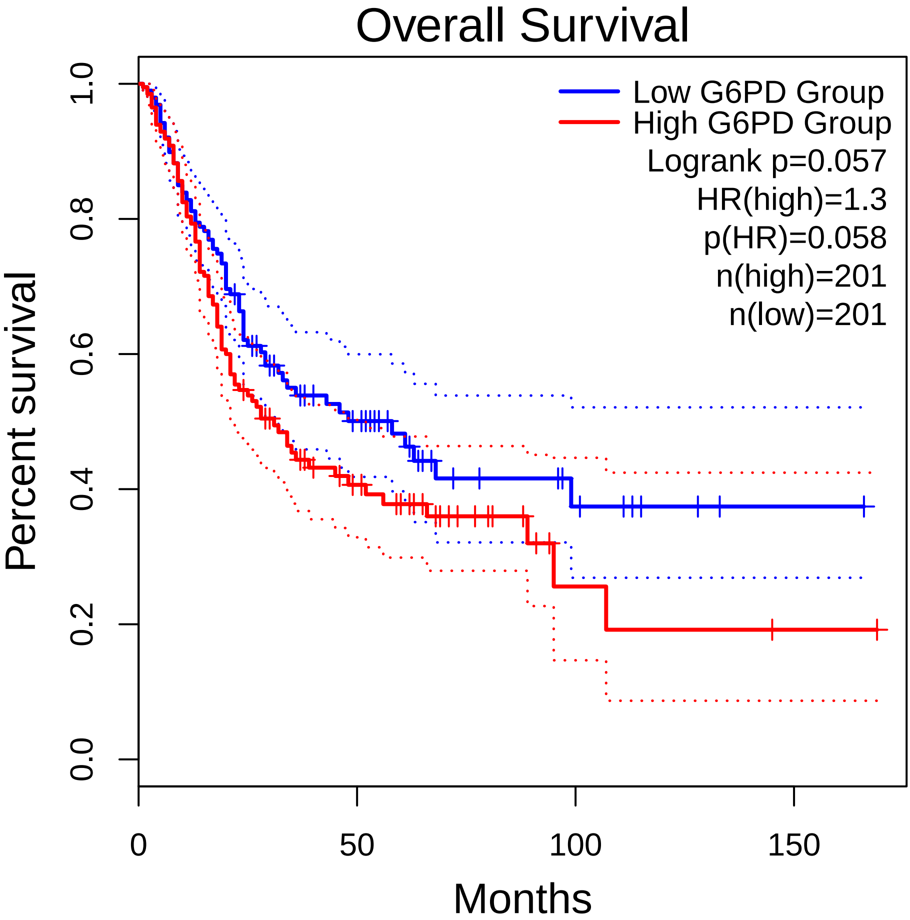

Overall Survival
Low G6PD Group
High G6PD Group
Logrank p=0.057
HR(high)=1.3
p(HR)=0.058
n(high)=201
n(low)=201
Percent survival
0.0
0.2
0.4
0.6
0.8
1.0
0
50
100
150
Months

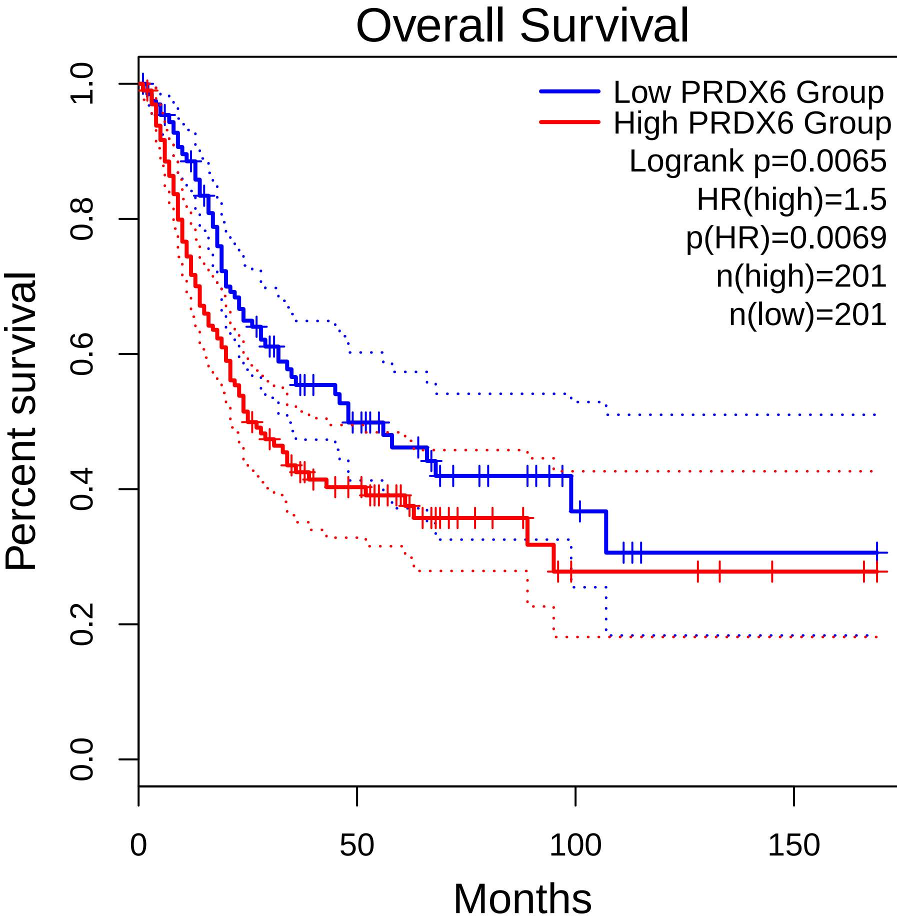

Overall Survival
Low PRDX6 Group
High PRDX6 Group
Logrank p=0.0065
HR(high)=1.5
p(HR)=0.0069
n(high)=201
n(low)=201
Percent survival
0.0
0.2
0.4
0.6
0.8
1.0
0
50
100
150
Months

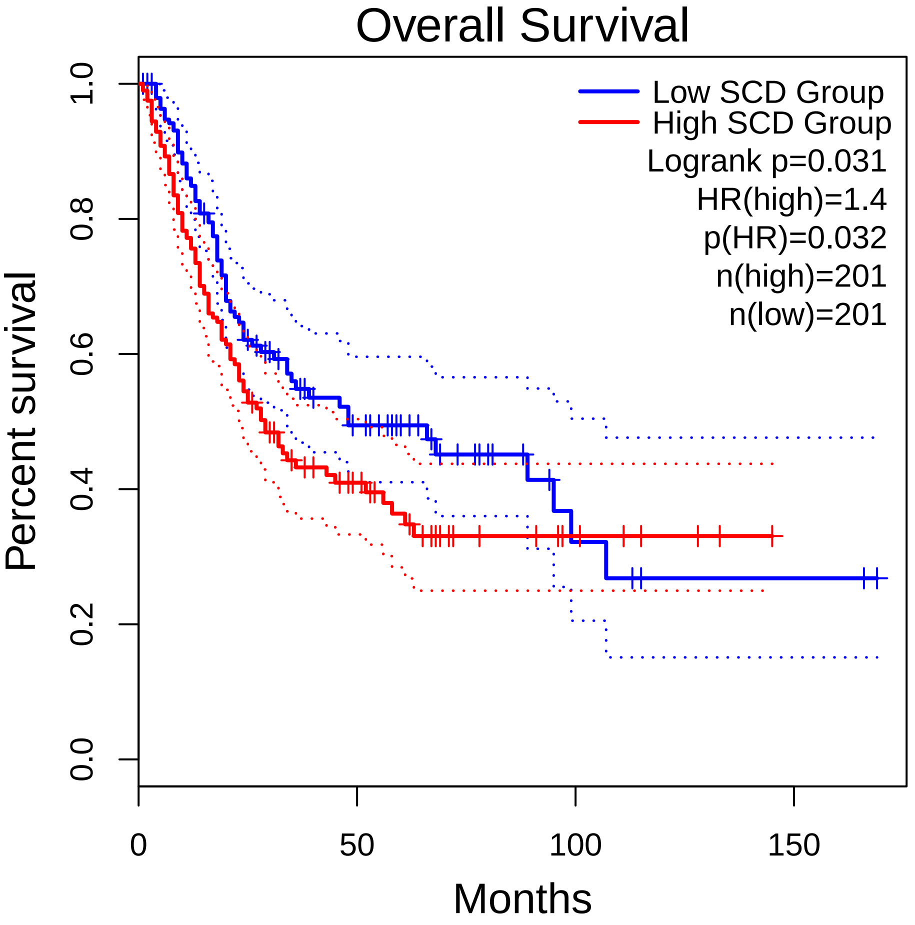
Overall Survival
Low SCD Group
High SCD Group
Logrank p=0.031
HR(high)=1.4
p(HR)=0.032
n(high)=201
n(low)=201
Percent survival
0.0
0.2
0.4
0.6
0.8
1.0
0
50
100
150
Months

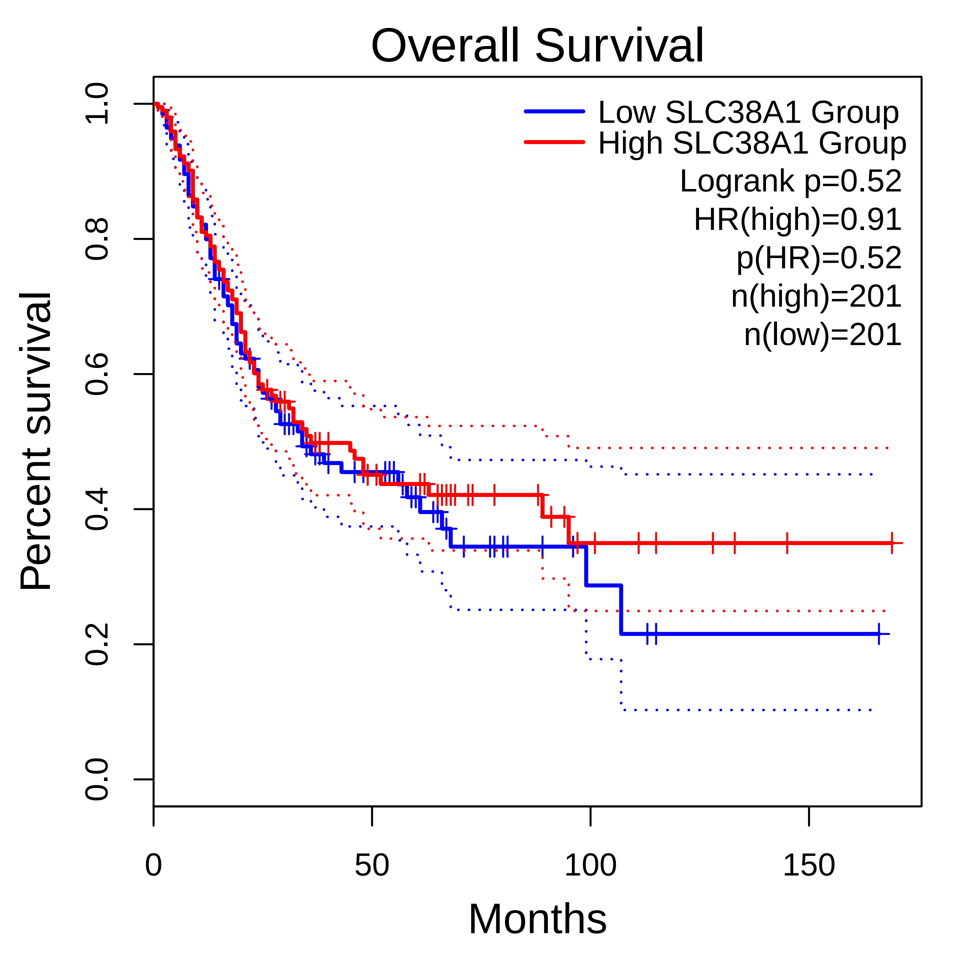
Overall Survival
Low SLC38A1 Group
High SLC38A1 Group
Logrank p=0.52
HR(high)=0.91
p(HR)=0.52
n(high)=201
n(low)=201
Percent survival
0.0
0.2
0.4
0.6
0.8
1.0
0
50
100
150
Months

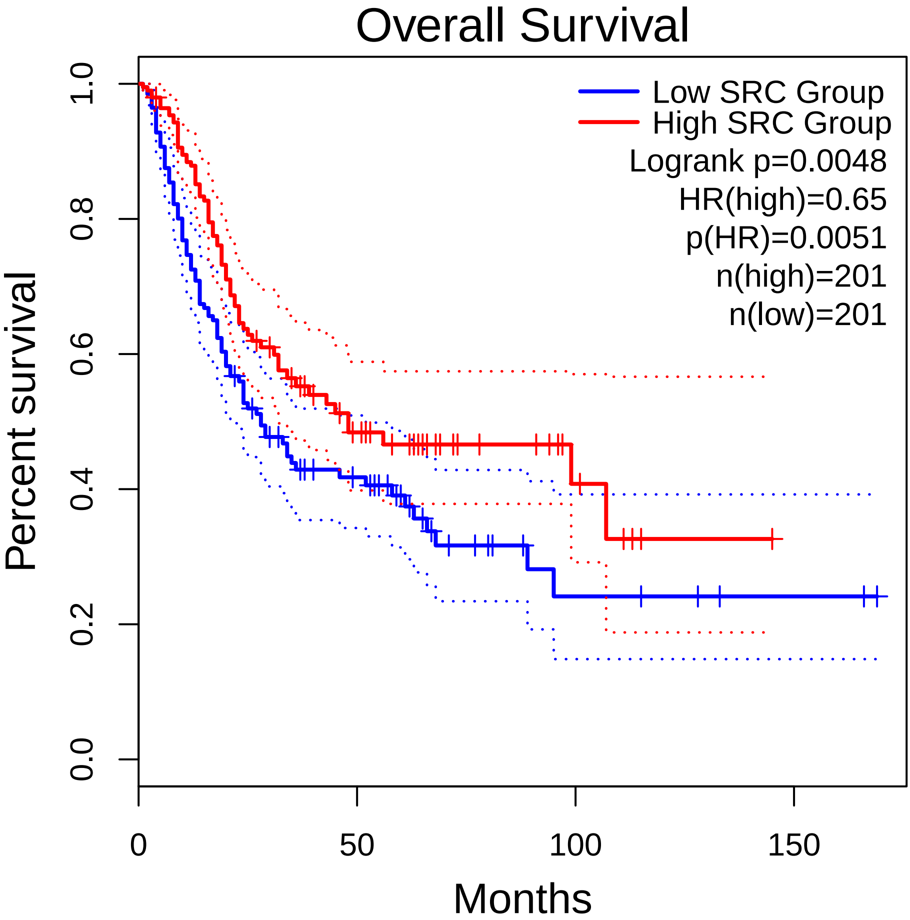

Overall Survival
Low SRC Group
High SRC Group
Logrank p=0.0048
HR(high)=0.65
p(HR)=0.0051
n(high)=201
n(low)=201
Percent survival
0.0
0.2
0.4
0.6
0.8
1.0
0
50
100
150
Months

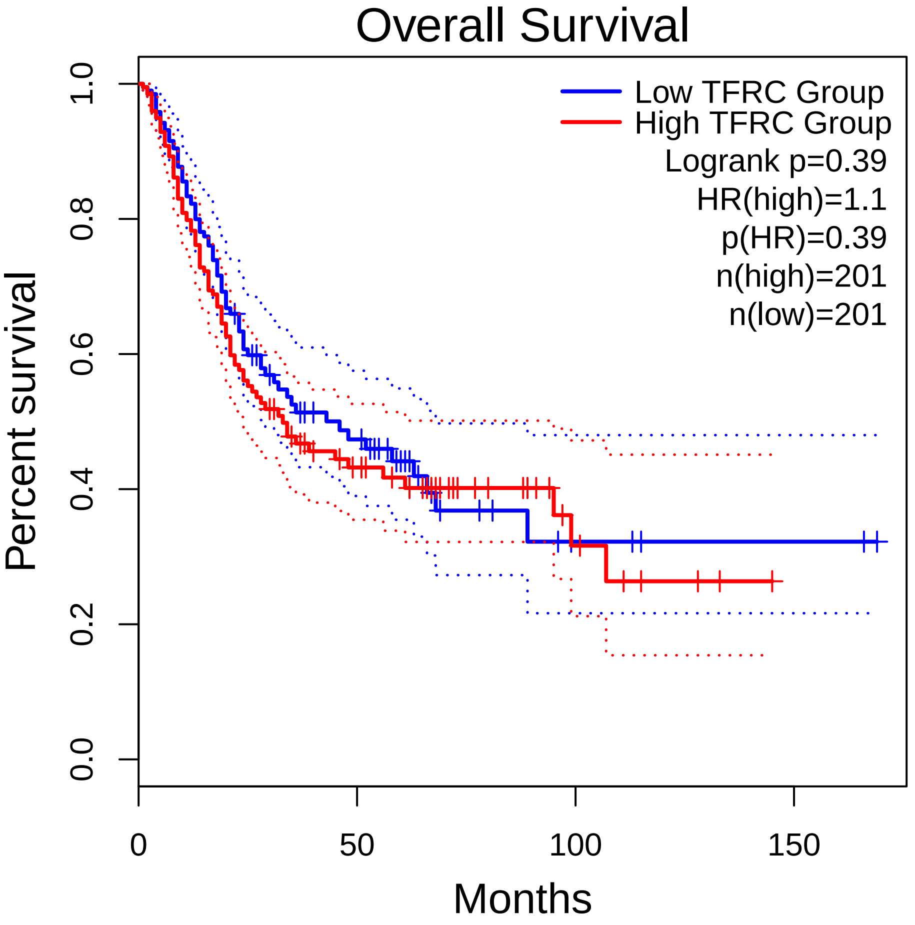

Overall Survival
Low TFRC Group
High TFRC Group
Logrank p=0.39
HR(high)=1.1
p(HR)=0.39
n(high)=201
n(low)=201
Percent survival
0.0
0.2
0.4
0.6
0.8
1.0
0
50
100
150
Months

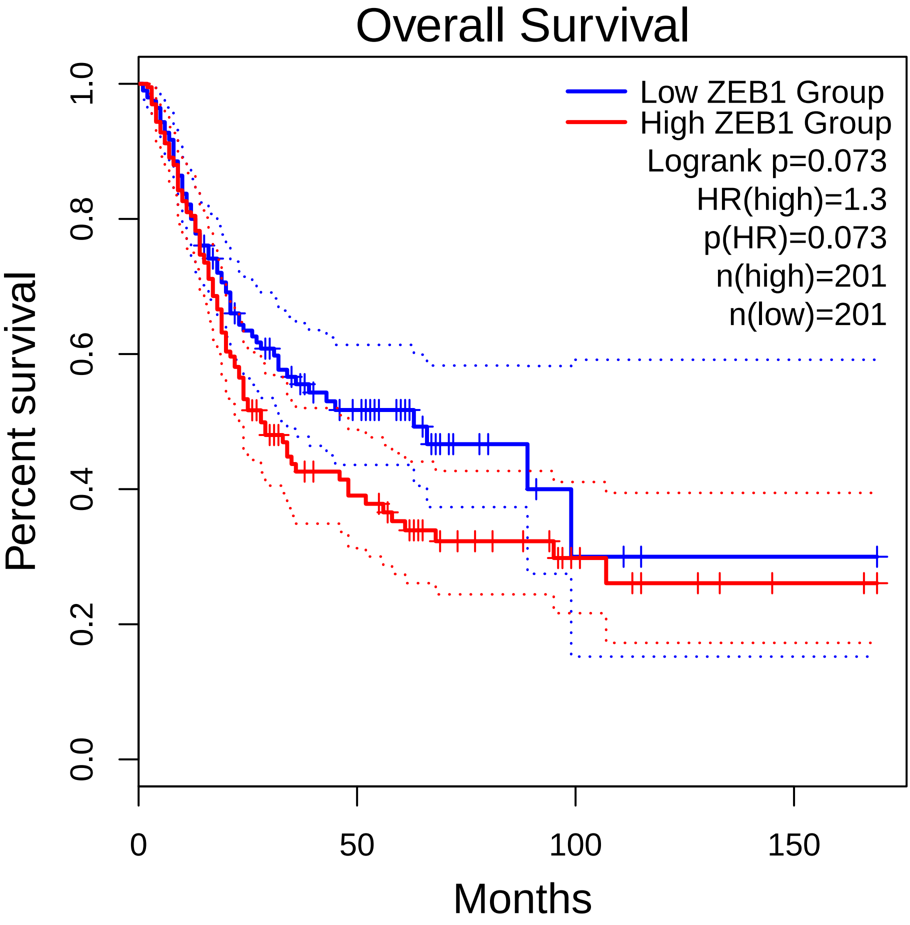
Overall Survival
Low ZEB1 Group
High ZEB1 Group
Logrank p=0.073
HR(high)=1.3
p(HR)=0.073
n(high)=201
n(low)=201
Percent survival
0.0
0.2
0.4
0.6
0.8
1.0
0
50
100
150
Months
