# Supplementary figures and images for: Identification of a Novel Ferroptosis-Related Gene Prognostic Signature in Bladder Cancer
Source: Front Oncol. 2021 Sep 7;11:730716. doi: 10.3389/fonc.2021.730716 (PMC8455063; doi:10.3389/fonc.2021.730716)

# G6PD

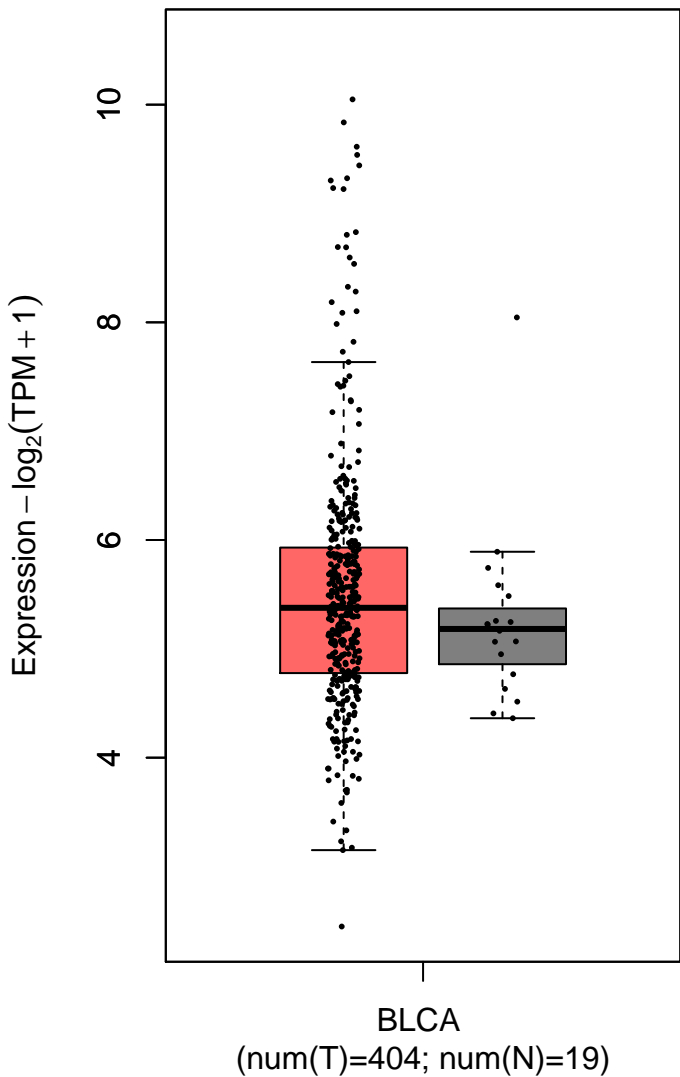

# PRDX6

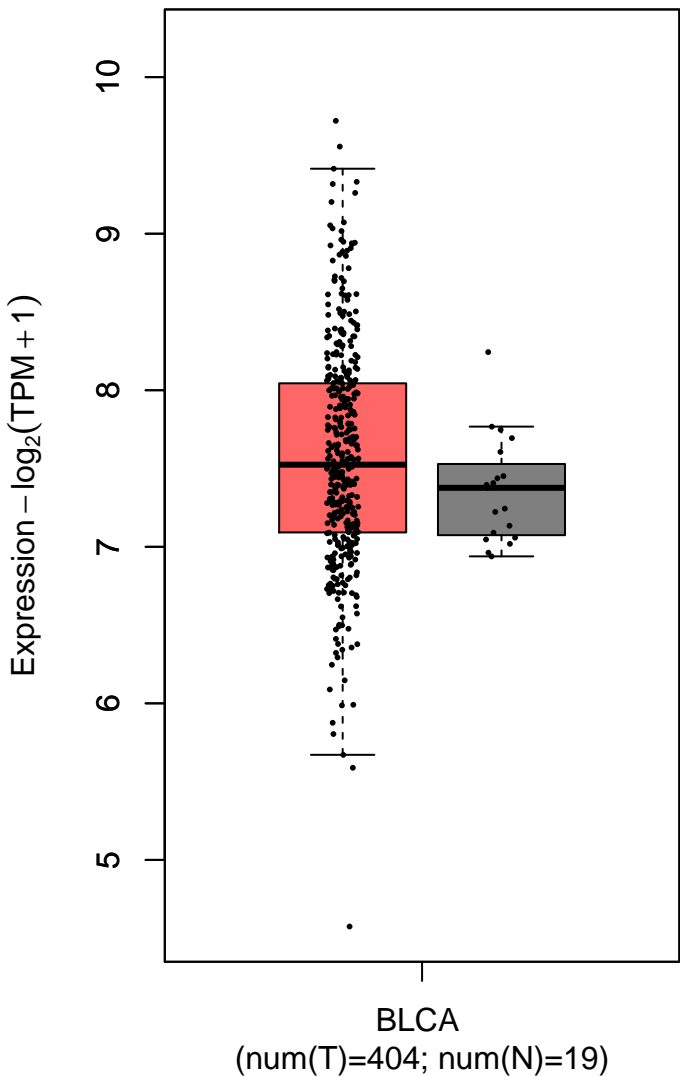

SCD

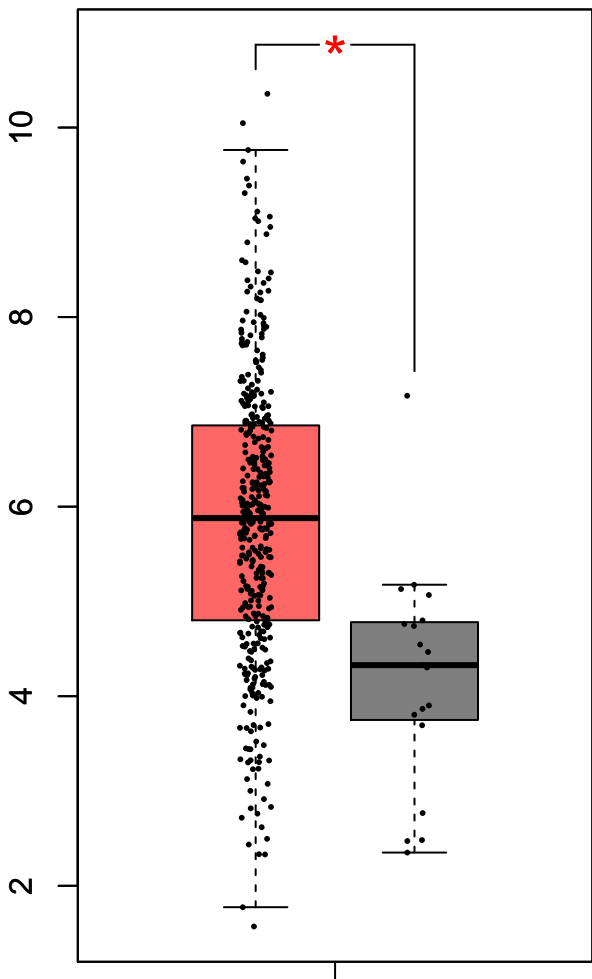

Expression - log<sub>2</sub>(TPM + 1)

BLCA

(num(T)=404; num(N)=19)

# SLC38A1

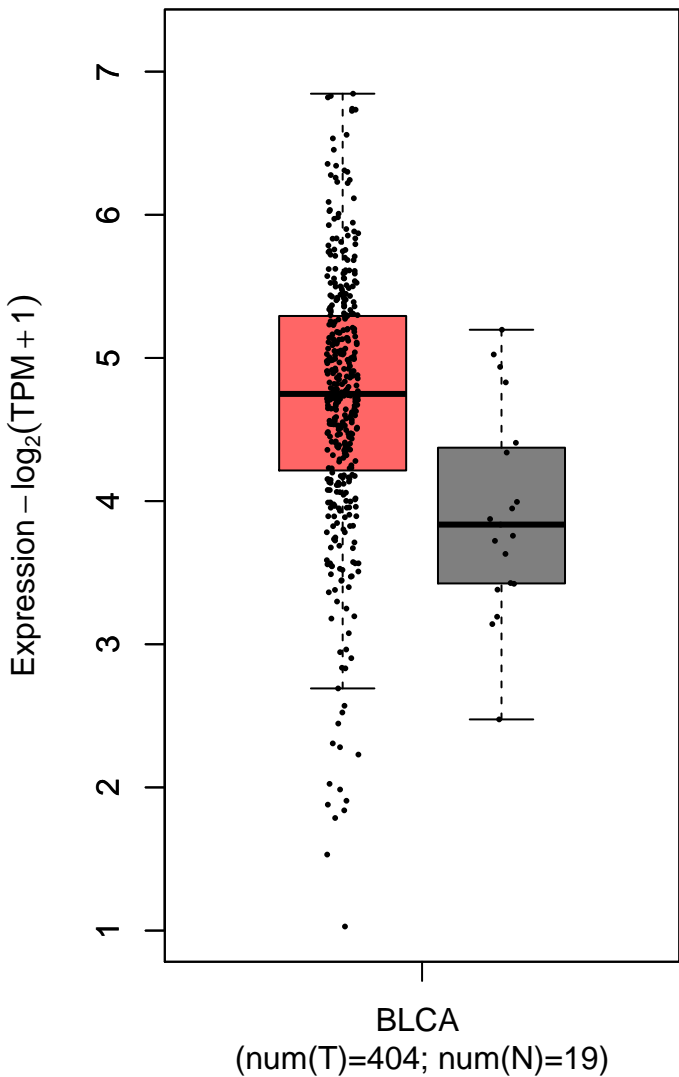

SRC

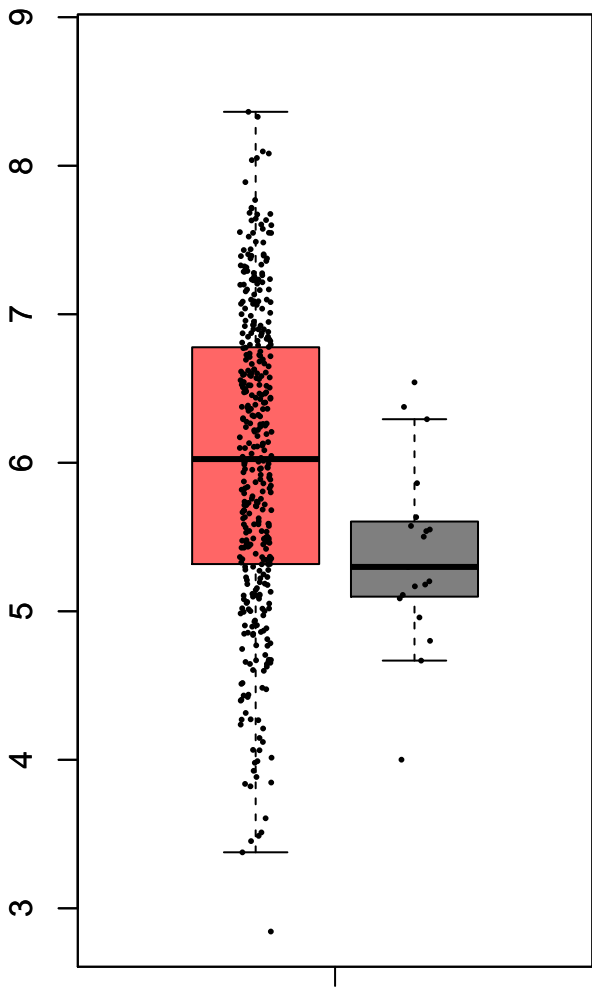

BLCA

(num(T)=404; num(N)=19)

# TFRC

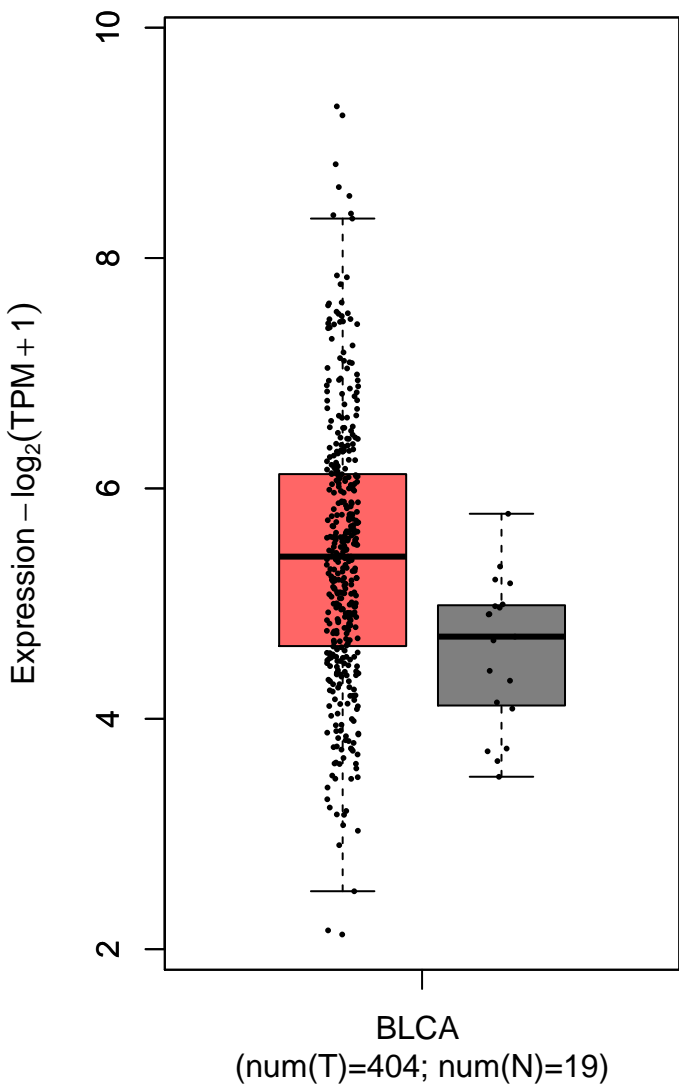

# ZEB1

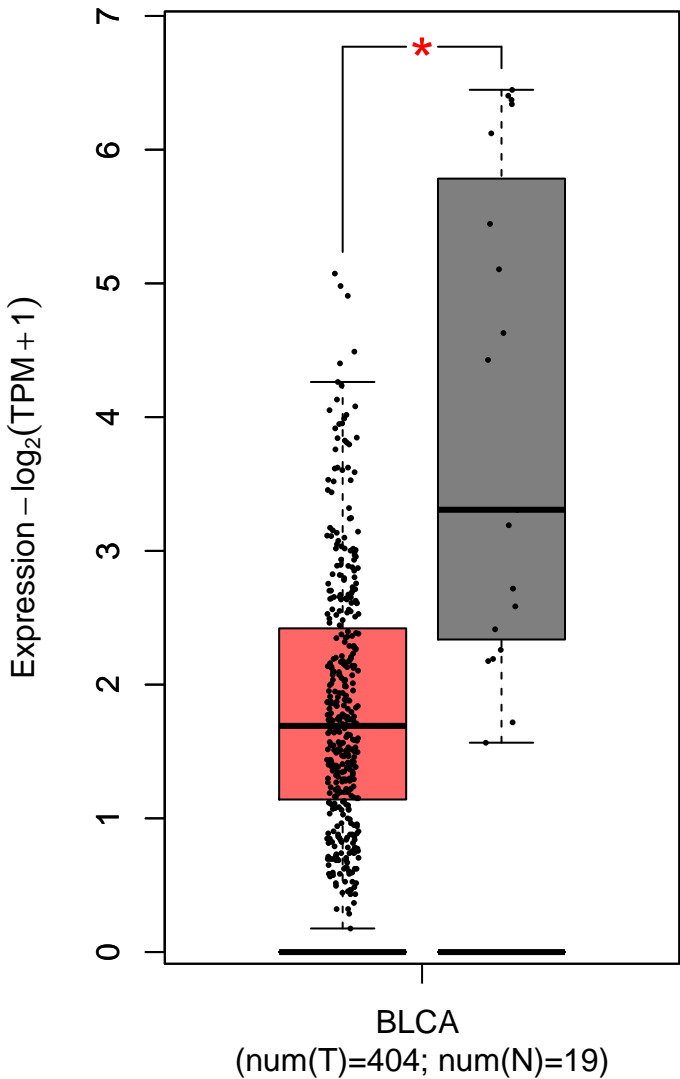

Supplement: Supplementary 2 — Significantly different FRG expression levels in BLCA and normal tissue in TCGA cohort. FRG, ferroptosis−related gene; TCGA, The Cancer Genome Atlas; BLCA, bladder cancer. [file DataSheet_2.pdf]

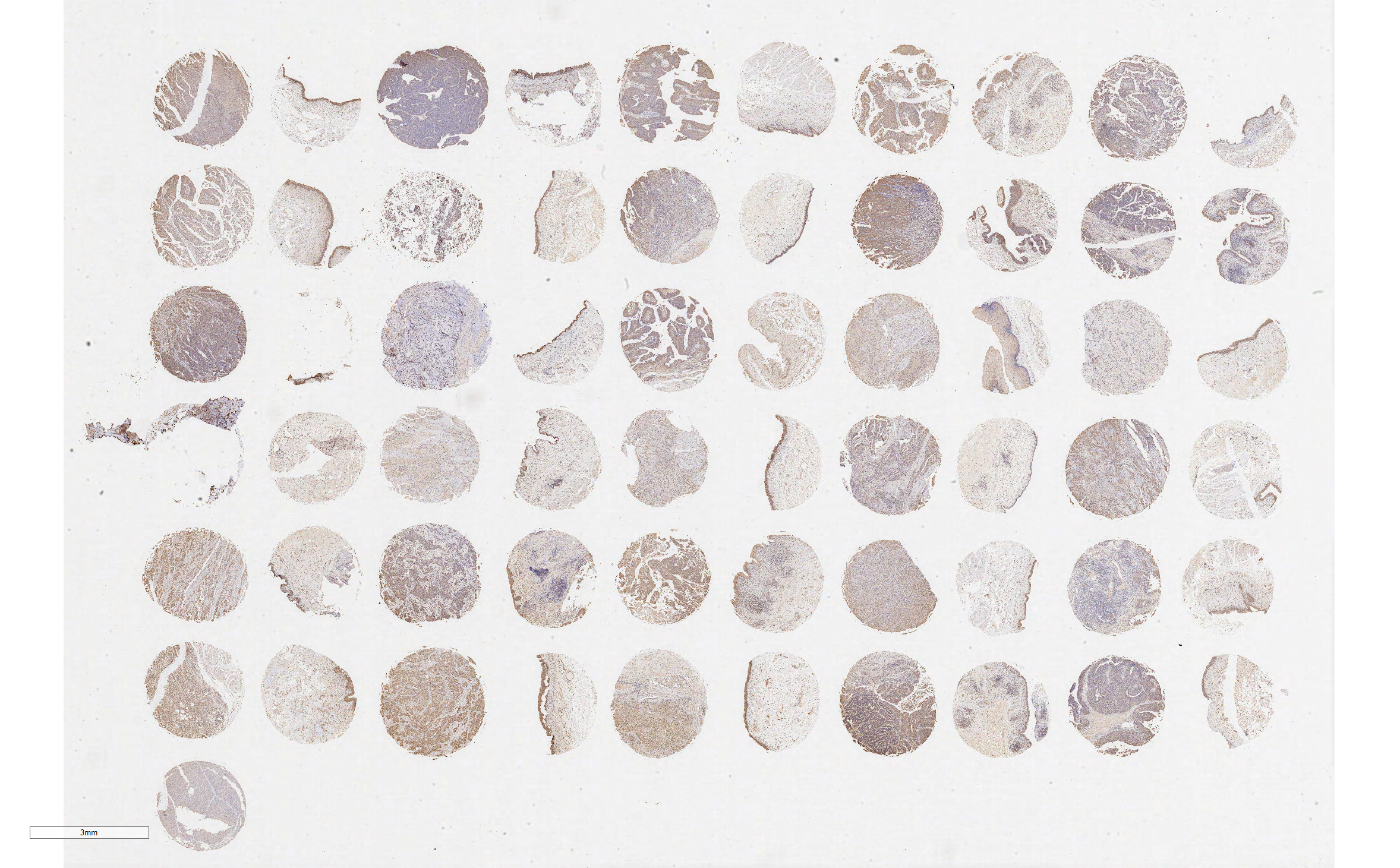

Supplement: Supplementary 3 — Boxplots of all genes in our FRG signature. FRG, ferroptosis−related gene. [file Image_1.tif]

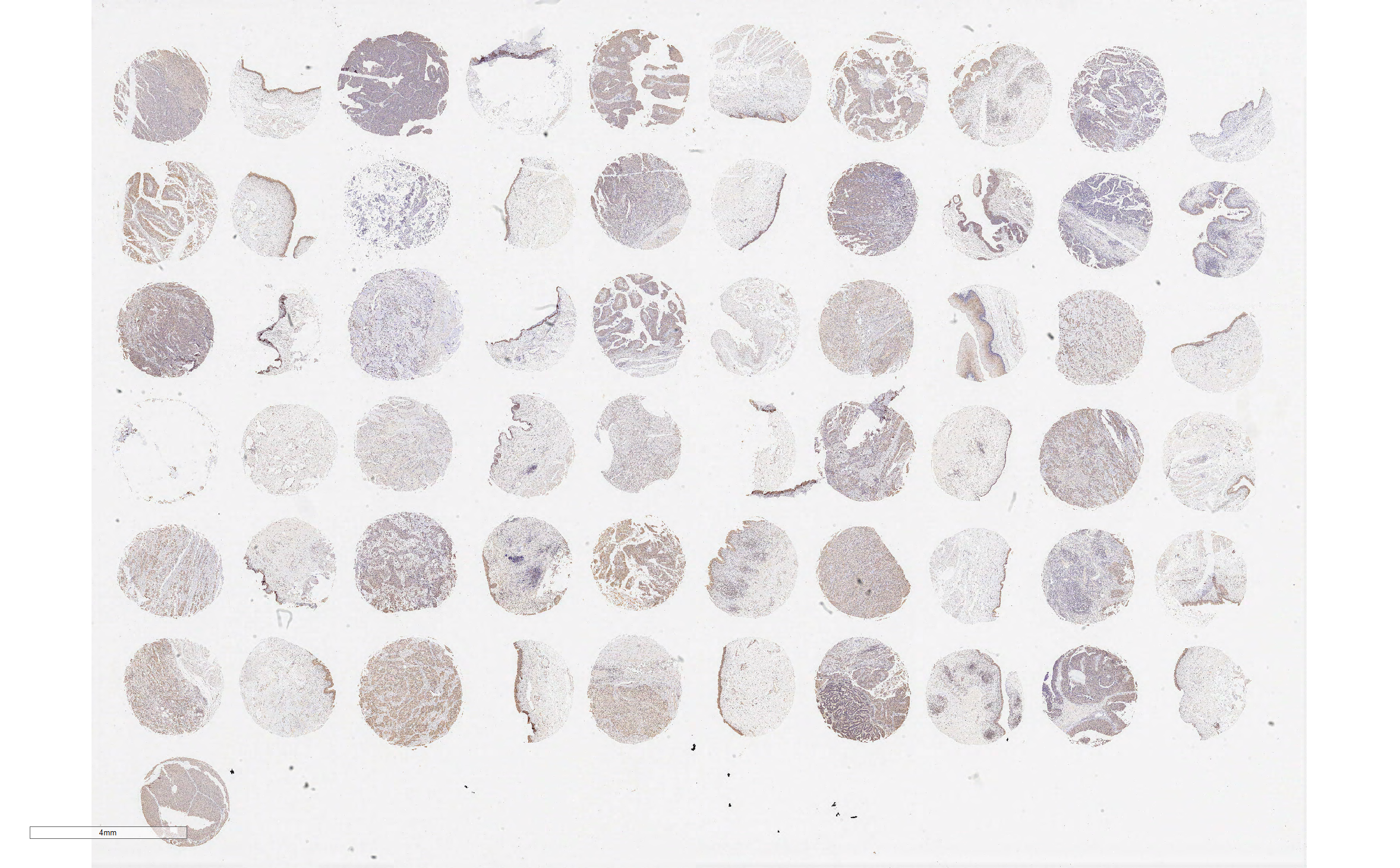

Supplement: Supplementary 4 — Survival curves of all genes in our FRG signature. FRG, ferroptosis−related gene. [file Image_2.tif]

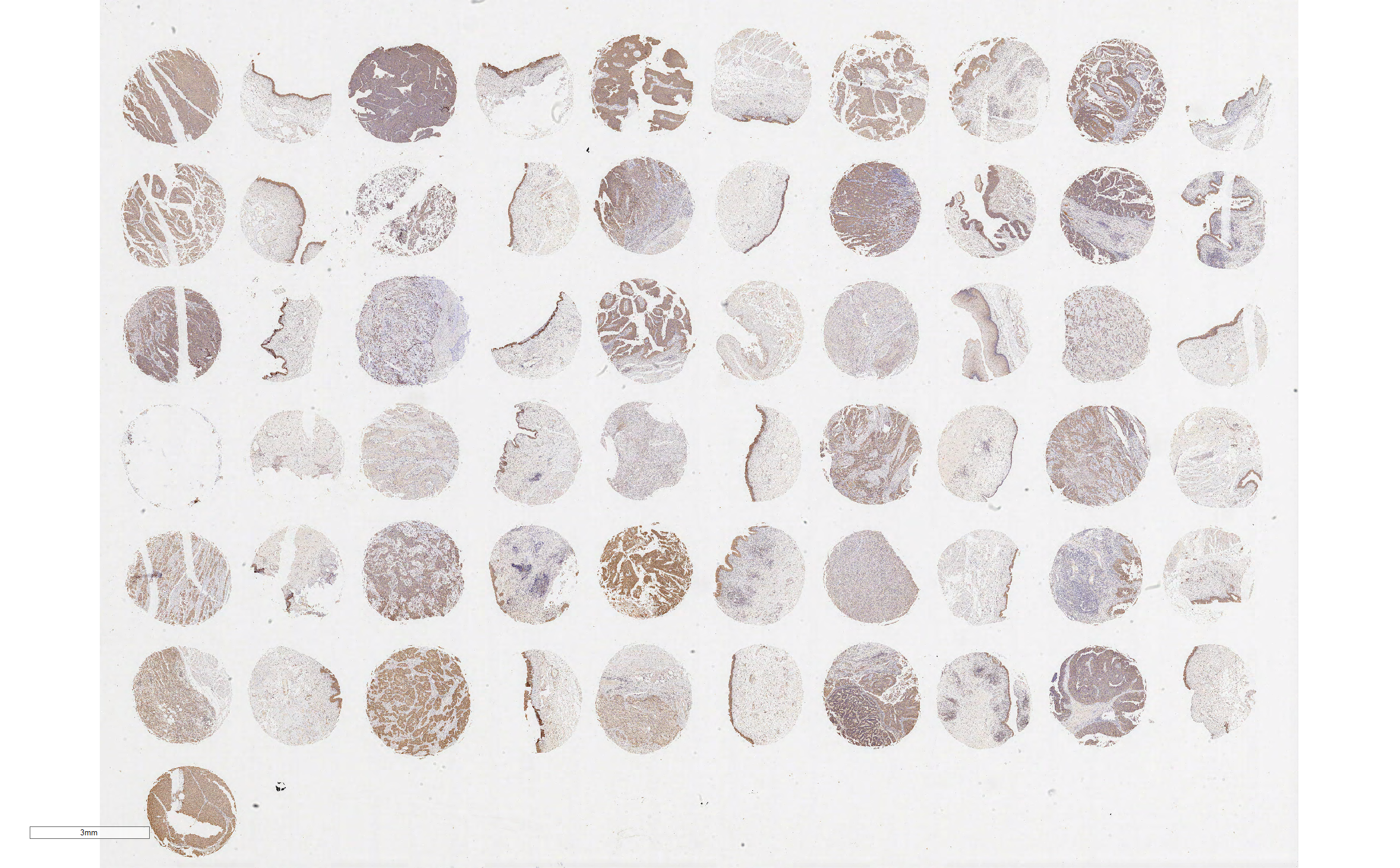

Supplement: Supplementary 5 — Detailed clinical information of patients with BLCA. [file Image_3.tif]
